# Extraction, Purification, and Biological Activities of Polysaccharides from Branches and Leaves of *Taxus cuspidata* S. et Z.

**DOI:** 10.3390/molecules24162926

**Published:** 2019-08-13

**Authors:** Ping Jiang, Qian Zhang, Yajie Zhao, Jia Xiong, Fei Wang, Ting Zhang, Chenmeng Zhang

**Affiliations:** 1 Jiangsu Key Lab of Biomass-Based Green Fuels and Chemicals, College of Chemical Engineering, Nanjing Forestry University, Nanjing 210037, China; 2 Plants for Human Health Institute, Food Bioprocessing and Nutritional Sciences, North Carolina State University, North Carolina Research Campus, Kannapolis, NC 28081, USA

**Keywords:** *Taxus cuspidata* branches and leaves, polysaccharides, extraction and purification, bioactivity

## Abstract

*Taxus cuspidata* S. et Z. is an excellent natural source of bioactive polysaccharides and has various biological activities. The objective of this study was to evaluate the effect of antidiabetic and antitumor activities of polysaccharides from *Taxus cuspidata* branches and leaves (TCBL) and to determine the optimum extraction technology of TCBL using a low-temperature and high-efficiency enzyme and ultrasound-assisted coupled extraction (EUCE) method. Optimal technology parameters were determined as follows: an extraction temperature of 51 °C, an extraction time of 33 min, a ratio of material to liquid of 1:19 (g:mL), and an enzyme concentration of 0.10 mg·mL^−1^. Under the optimized conditions, the polysaccharide yield from TCBL obtained by EUCE was 4.78% ± 0.18%. The four purified polysaccharides (Pe1, Pe2, Pe3, Pe4) from TCBL are mainly composed of arabinose, galactose, glucose, a small amount of xylose, and mannose. This composition was assessed by HPIC analysis. The antidiabetic activity and antitumor activity of polysaccharides from TCBL were assayed in vitro. Among the four purified polysaccharides from TCBL, purified Pe4 had the highest inhibitory capacity against α-glucosidase, and its IC_50_ value was 123.0 µg·mL^−1^. Pe1 had the highest antitumor capacity against MCF7 cells and HepG2 cells, with IC_50_ values of 169.0 and 132.0 µg·mL^−1^. Pe4 had the highest antitumor effect on human cervical cancer cells (Hela), and its IC_50_ value was 89.9 µg·mL^−1^. Pe4 polysaccharide demonstrated a good α-glucosidase inhibitory activity and antitumor capacity against Hela cells. Therefore, Pe4 polysaccharide from TCBL is a beneficial source of potential inhibitors of type II diabetes and human cervical cancer activity.

## 1. Introduction 

The genus *Taxus* is a member of the Taxaceae family, which includes 24 species [1]. The genus *Taxus* includes plant sources of good biologically and pharmacologically active compounds, especially the important broad-spectrum anticancer drug paclitaxel and many other taxane derivatives [2,3]. A total of 130 taxane diterpenoids, including paclitaxel and cephalomannine, have been isolated from *T. canadensis* over the past 30 years [4]. Moreover, the results of recent studies indicate that the plants of the genus *Taxus* contain many other biologically and pharmacologically active compounds in addition to taxane diterpenoids. Various fatty acids from *T. mairei* leaves are identified by GC–MS [4]. Commercially available yew alkaloid toxoids were discerned and quantified by LC–MS/MS [5]. Flavonoids and lignins were separated and purified from *Taxus* in recent years [6]. A novel heteropolysaccharide from *T. yunnanensis* leaves was separated and purified by anion-exchange chromatography and GPC [7]. 

Furthermore, the results of recent studies indicate that extracts and compounds extracted and isolated from *Taxus* possess significant anticancer, anti-inflammatory, antidiabetic, antifungal, antibacterial, and anticonvulsant activities, and so on. Aqueous extract from *T. chinensis* can usually inhibit the A549 cells in vivo and in vitro [8]. Aqueous extract of *T. mairei* might repress A549 xenograft growth [9]. In vitro studies have shown that the extract from *T. cuspidata* possessed broad-spectrum anticancer activity in different cancer cells lines such as HL-60, BGC-823, KB, Bel-7402, HeLa, and pancreatic cancer [10,11]. Aqueous extract from *T. baccata* retains significant inhibitory activity against the adenosine deaminase enzyme, so it might play an important role in the anticancer activities of *Taxus* species [12]. Alcoholic extract from *T. baccata* displays strong anti-inflammatory activities such as bronchodilation and decreased bronchial hyperreactivity [13,14]. In addition, it was previously reported that the compositions from *Taxus* have major effects on the cardiovascular system and the nervous system [15]. Seven compounds from *T. cuspidata* exhibit 5–13-fold stronger inhibitory activity against U46619-induced aggregation than acetylsalicylic acid (ASA). Because of the inhibitory capacity against both butyrylcholinesterase and lipoxygenase (LOX), lignans from *T. baccata* play a vital role in the pathogenesis of Alzheimer’s disease [16]. Recent reports indicate that the polysaccharides extracted and separated from *Taxus* possess potent antitumor and antidiabetic properties. The polysaccharides from *T. cuspidata* exhibited antidiabetic properties in diabetic mice [17]. A polysaccharide from *T. yunnanensis* showed a greater inhibitory effect on MCF-7 and TMP70W cells [7]. PSY-1 polysaccharide from *T. mairei* leaves exhibited antineoplastic effects on the melanoma cancer cell line B16-F10 [18]. Polysaccharides from *Taxus chinensis* var. *mairei* fruits have the inhibitory effect on S180 tumor growth in vivo [19].

The extraction methods of polysaccharides include heating reflux [20], ultrasonic-assisted extraction [21,22], and microwave-assisted extraction [23,24]. There are some weaknesses in these extraction methods, such as lower extraction yields, longer extraction times, and higher extraction temperatures, respectively. Polysaccharides from *Taxus chinensis* fruits were extracted using an ultrasonic/microwave coupled extraction method by Zhao et al. Under optimum conditions, the yield of polysaccharides was 4.33% [19].

Extracting active substances at a lower temperature is the main advantage of the enzymatic extraction approach. Ultrasound-assisted extraction can improve mass transfer, intensify cell disruption, and accelerate solvent penetration effects [25]. Therefore, a novel and efficient enzyme and ultrasound-assisted coupled extraction method of polysaccharides from *Taxus cuspidata* branches and leaves (TCBL), which can not only improve the extraction process but also increase the extraction efficiency of natural compounds [26], was explored in this study. The enzyme and ultrasound-assisted coupled extraction method is a complementary extraction technique. Although some reports have appeared about the optimization of the extraction technique of polysaccharides, studies regarding the enzyme and ultrasound-assisted coupled extraction of polysaccharides from TCBL are few in number. 

In this study, the extraction technology of enzymatic and ultrasound-assisted coupled extraction of polysaccharides from TCBL was optimized. The purified polysaccharides were obtained by separating and purifying with macroporous adsorption resin, dialysis, DEAE cellulose column chromatography, and an alcohol precipitation method of different ethanol concentrations. The antidiabetic properties of polysaccharides from TCBL were evaluated with an in vitro assay by inhibiting α-glucosidase activity. The inhibition effects of purified polysaccharides from TCBL on MCF7, HepG2, and Hela cells were carried out to evaluate the antitumor properties with an in vitro assay.

## 2. Materials and Methods

### 2.1. Chemicals and Solvents

Analytical-grade reagents were purchased for this study: p-nitrophenyl-α-d-glucopyranoside (PNPG), tocopherol, hydroxybiphenyl, α-glucosidase (14.5 U/mg), acarbose, PBS (Sigma, Shanghai, China), ethanol, petroleum ether, methanol, DMSO, methanol (HPLC grade), 98% sulfuric acid, anthrone, sodium chloride, phosphate buffer, sodium carbonate, and sodium hydroxide (Nanjing Chemical Reagent Co., Ltd., China). AB-8 macroporous adsorption resin was purchased from Bengbu Liaoyuan New Materials Co., Ltd. Anhydrous glucose, DEAE cellulose, and dialysis bags were purchased from Shanghai Yuanye Biotechnology Co., Ltd. Pectinase was purchased from Kandin Chemical Reagent Co., Ltd. (Guangzhou, China). DMEM was purchased from Thermo Fisher Scientific Instrument Co., Ltd. (Waltham, MA, USA).

### 2.2. Plant Material

The fresh raw *Taxus cuspidata* branches and leaves (TCBL) were collected in July 2017 in Rongcheng, China. The TCBL materials were cut into pieces with scissors and air-dried. The dried branches and leaves were ground to particles and passed through a 425 μm sieve using a multifunction pulverizer (Yongkang, China), with a moisture content of 6.33% for reserve.

### 2.3. Equipment

Equipment included an electronic balance (AY120 Beijing Sedoris Instruments Co., Ltd.); SHB-IIIG vacuum pump (Zhengzhou Great Wall Technology Industry and Trade Co., Ltd.); UV-2450 ultraviolet-visible spectrophotometer (SHIMADZU Company of Japan); AC5200DTD ultrasound cleaning machine (Nanjing Anxiu Instrument Equipment Co., Ltd.); TDZ5-WS centrifuge (Changsha Xiangzhi Centrifuge Instrument Co., Ltd.); HH-2Water bath pot (Changzhou Tianrui Instrument Co., Ltd.); microplate reader (Cytation3 BioTek, Beijing China); magnetic stirrer (CL-200 Gongyi City Yuhua Instrument Co., Ltd.); freeze dryer (FDV-1200 Shanghai Ailang Instrument Co., Ltd.); and ion chromatograph (ICS-5000 Thermo Fisher Scientific Instrument Co., Ltd.).

### 2.4. Experimental Methods

#### 2.4.1. Enzyme and Ultrasound-Assisted Coupled Extraction (EUCE) Single-Factor Experiment 

The powdered TCBL samples (3.0 g) were dissolved in the pectinase enzyme solution, and ultrasound-assisted extraction was carried out. The influencing factors of different extraction temperatures, ratios of raw material to liquid, extraction times, and enzyme concentrations were investigated with the enzyme and ultrasound-assisted coupled extraction (EUCE) method. The effect of the material to liquid ratio (1:14, 1:16, 1:18, 1:20, and 1:22 g:mL), extraction time (20, 30, 40, and 50 min), extraction temperature (30, 40, 50, and 60 °C), enzyme concentration (0.050, 0.075, 0.100, and 0.125 mg·mL^−1^), and ultrasonic power (96, 120, 144, 168, and 192 W) were all investigated using a single-factor design. The obtained extraction solutions were filtered and transferred to a 50 mL volumetric flask and filled up to the volume. All assays were repeated twice.

#### 2.4.2. Enzyme and Ultrasound-Assisted Coupled Extraction (EUCE) Response–Surface Optimization

On the basis of single-factor experiment results, a response–surface optimal experimental design was performed by the Box–Behnken model of Design–Expert software (v8.0.6.1, Minneapolis, MN, USA). The extraction temperature, enzyme concentration, extraction time, and ratio of raw material to liquid were selected to design 4 factors and 3 levels of response–surface experiments. The coefficients of the polynomial model and optimum extraction technology were obtained from the experimental design using Design-Expert 8.0.6 software.

#### 2.4.3. Total Polysaccharide Yield Calculation

The aqueous extract of *Taxus* was diluted to a certain multiple, and 2 mL of the diluted solution was taken. The absorbance of the solution was measured by the anthrone–sulfuric acid method [27]. The total polysaccharide mass concentration was calculated according to the glucose standard curve, and the total polysaccharide yield of *Taxus cuspidata* branches and leaves was calculated from the following formula (1):

(1) $$Y(\%)=\frac{C\times V\times n\times 0.9}{G\times (1-w)}\times 100\%$$

where *Y* (%) is the polysaccharide yield, *C* is the mass concentration of the extraction solution (mg·mL^−1^), *V* is the volume of the extraction solution (mL), n is the dilution factor, *G* is the weight of the power raw material (g), and w is the moisture content of the raw material (%). 

Taking the absorbance (A) as the ordinate and the anhydrous glucose concentration (C) as the abscissa to draw the glucose standard curve, the regression equation of the standard curve is A = 9.978C − 0.0009 (R^2^ = 0.9995), and the linear range is 0.01–0.1 mg·mL^−1^. The mass concentration *C* of polysaccharide was calculated according to the standard curve.

#### 2.4.4. Pretreatment of Raw Materials by Preparation of Polysaccharides

The TCBL raw materials were degreased 3 times with petroleum ether. Then, they were cold-soaked 3 times with 80% ethanol for 24 h, filtered, and air-dried to remove ethanol.

#### 2.4.5. Extraction of the Polysaccharides

The polysaccharides from the pretreated TCBL materials were extracted in triplicate by enzyme and ultrasonic-assisted extraction at a temperature of 51 °C, time of 33 min, ratio of material to liquid of 1:19 g·mL^−1^, and enzyme concentration of 0.097 mg·mL^−1^. The polysaccharide extract was obtained by evaporating under reduced pressure. 

#### 2.4.6. Separation and Purification of Polysaccharides from the Polysaccharide Extract

The crude polysaccharide extract of TCBL contained more protein, pigments, and other substances; therefore, it was necessary to carry out the separation process to remove them by the macroporous adsorption resin method. The crude polysaccharides were further purified using an alcohol precipitation method. When different concentrations of ethanol precipitation solutions were refrigerated for 24 h and centrifuged at 3500 r/min for 15 min, four crude polysaccharides of TCBL (50%, 60%, 70%, and 80% alcohol precipitation polysaccharides) were obtained by freeze drying, respectively.

The polysaccharide obtained by alcohol precipitation method also contained a small amount of monosaccharide and some small molecular substances, which were further purified by dialysis and DEAE cellulose column chromatography. Freeze-dried 50% alcohol-precipitated polysaccharide (Pe1), 60% alcohol-precipitated polysaccharide (Pe2), 70% alcohol-precipitated polysaccharide (Pe3), and 80% alcohol-precipitated polysaccharide (Pe4) were obtained, respectively.

#### 2.4.7. Analysis of Monosaccharide Composition of Polysaccharides from TCBL

Samples of polysaccharides from TCBL underwent an acid hydrolysis method in order to prepare different monosaccharide compositions. The analysis of samples that contained monosaccharide was conducted on ICS-5000 HPIC (Thermo fisher company, USA) using a Dionex Carbo Pac PA 200 column (3 × 250 mm) equipped with a guard column, Dionex CarboPac PA200 column (3 × 50 mm). The mobile phase consisted of 10 mM NaCl solution. The operating conditions were as follows: column oven at 30 °C, constant elution flow rate of 0.5 mL/min, and an injection volume of 5 μL (0.2 mg·mL^−1^ samples of arabinose, glucose, galactose, xylose, mannose, and hydrolyzed *Taxus* polysaccharides).

#### 2.4.8. α-Glucosidase Inhibitory Activity of Polysaccharides from TCBL

The methods for α-glucosidase inhibitory activity assays were adapted from Stephen et al. [28]. For α-glucosidase inhibitory activity assays, 10 μL sample of different concentrations or a positive control were added to 120 μL of a 0.1 U/μL α-glucosidase phosphate buffer solution in a 96-well plate and incubated at 37 °C for 10 min. In total, 20 μL of a 2.5 M PNPG solution was added to each well and incubated at 37 °C for another 15 min. Finally, 80 μL of a 0.2 M NaCO_3_ solution was added immediately before reading the absorbance at 405 nm. The current diabetes drug acarbose as the positive control demonstrated 100% inhibition.

The inhibitory activity of the different samples of polysaccharides from TCBL was calculated by the following equation:

Inhibitory activity % = (A_2_ − (A_1_ − A_0_))/A_2_ × 100% (2)

where A_0_ is the absorbance value of the sample solution at different concentrations, A_1_ is the absorbance value of the α-glucosidase with the samples, and A_2_ is the absorbance value of the α-glucosidase solution without the inhibitor.

#### 2.4.9. Antitumor Activity Study of Polysaccharides from TCBL

Three tumor cells (human liver cancer (HepG2), human cervical cancer (Hela), and human breast cancer (MCF7)) were used in the antitumor activity assays, which were provided by the Institute of Botany, Chinese Academy of Sciences, Jiangsu Province. Three tumor cells in the logarithmic growth phase were inoculated in 96-well plates at a density of 5000 cells per 100 μL and incubated for an additional 24 h. Then, 100 μL of the samples of different concentrations were added to each well. The plate was incubated at 37 °C for 72 h in the incubator with 5% CO_2_/95% air. Ten microliters of 5 mg·mL^−1^ MTT was placed in each well and incubated in an incubator for 4 h. Afterward, the supernatant was removed, and 100 μL of DMSO was added to each well in the 96-well plates to dissolve the solid crystal. The absorbance was read at 490 nm. The current antitumor etoposide was used as the positive control. The cell growth inhibition rate was calculated according to Formula (3), and the IC_50_ value was obtained.

Inhibition rate % = (A_2_ − A_1_)/A_2_ × 100% (3)

where A_1_ is the absorbance value after the samples inhibited growth of the tumor cells, and A_2_ is the absorbance value of the blank control.

#### 2.4.10. Statistical Analyses

Optimum extraction technology conditions were repeated for the test twice. Data of bioactivity assays of polysaccharides from TCBL were expressed as means ± SD of independent duplicates with three replications. Statistical analyses were conducted as means ± SD by Origin 8 software. The IC_50_ values were received from sample solutions with different concentrations. Significant differences were indicated by *p* < 0.05. 

## 3. Results and Discussion

### 3.1. Comparison of Extraction Methods of Polysaccharides from Taxus cuspidata Branches and Leaves (TCBL) 

During the extraction process, as a biocatalyst, the enzyme decomposes plant tissue under mild conditions, destroys the dense structure of the cell wall, and accelerates the release and extraction of the active ingredient. The pectinase enzyme has the function of hydrolyzing pectin. The high efficiency of the enzyme can save energy and time. The specificity and selectivity of the enzyme make the product stable, high purity, high activity. 

The experimental results are shown in Table 1. The polysaccharide yield by EUCE was the highest among the four extraction methods. The polysaccharide yield by EUCE was 4.47% ± 0.02% and increased by 20.49%, 14.91%, and 4.68% compared to that of hot water reflux extraction (HWRE), ultrasonic-assisted extraction (UAE), and enzyme extraction (EE), respectively. Therefore, the EUCE method for polysaccharide extraction from *Taxus cuspidata* branches and leaves was the superior extraction method.

### 3.2. The Results of Single-Factor Experiments of Extraction Technology of Polysaccharide from TCBL

#### 3.2.1. Effect of Extraction Temperature on Polysaccharide Yield from TCBL

**Figure 1:**
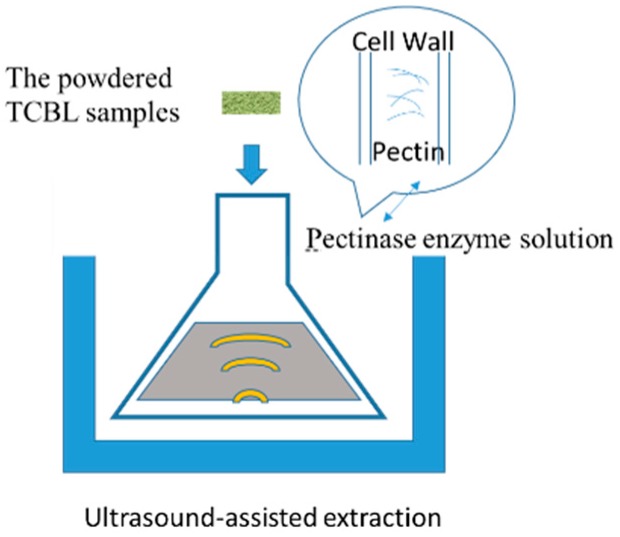
Enzyme and ultrasound-assisted coupled extraction.

Three grams of crushed material from *Taxus cuspidata* branches and leaves (TCBL) was extracted at different temperatures of 30, 40, 50, and 60 °C, with a ratio of material to liquid of 1:16 (solution of 0.100 mg·mL^−1^ pectinase concentration) and ultrasonic treatment of 144 W for 40 min. The effect of extraction temperature on TCBL polysaccharide yield was investigated by the EUCE method. The result is shown in Figure 1.

**Figure 2:**
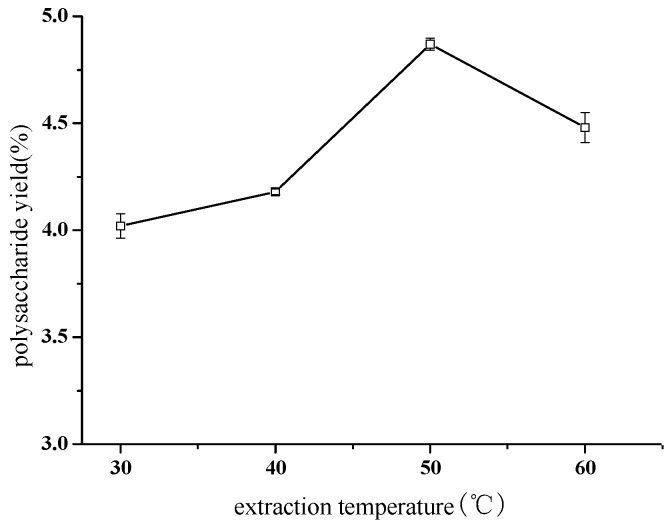
The effect of extraction temperature on polysaccharide yield (w/w dry crushed material %) from TCBL.

As shown in Figure 2, the yield of polysaccharides from TCBL increased with the elevation of the extraction temperature in the temperature range of 30 to 50 °C. The highest yield of TCBL polysaccharides was 4.87% at 50 °C. The yield of TCBL polysaccharides decreased when the temperature was higher than 50 °C. With the increase in the extraction temperature, the extraction yield of polysaccharides of *Taxus* increased. When the concentration of the internal and external cells of the tissue reached equilibrium and the extracted polysaccharide reached the equilibrium of dissolution, the extraction yield of polysaccharide began to decrease. When the temperature was too high, the enzyme protein desaturated, thereby weakening the enzyme activity. The reaction rate decreased rapidly with an increase in temperature; therefore, the extraction yield of polysaccharide decreased. As the figure shows, the optimal temperature to obtain the greatest extraction yield of *Taxus* polysaccharide was 50 °C.

#### 3.2.2. Effect of Extraction Time on Polysaccharide Yield from TCBL

**Figure 3:**
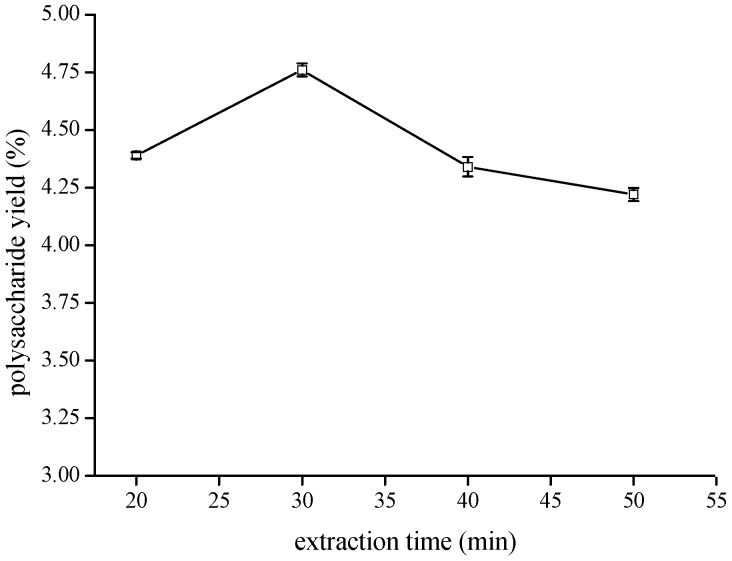
Effect of extraction time on polysaccharide yield (w/w dry crushed material %) from TCBL.

A quantity of 3 g of crushed material from *Taxus cuspidata* branches and leaves (TCBL) was extracted at different times of 20, 30, 40, and 50 min, a ratio of material to liquid 1:16 (solution of 0.100 mg·mL^−1^ pectinase concentration), and ultrasonic treatment of 144 W at 50 °C. The effect of the extraction time on TCBL polysaccharide yield was investigated by the EUCE method. The result is shown in Figure 3.

The extraction time is an important influencing factor regarding the extraction of TCBL materials as solvent penetrated into the material cell and dissolved ingredients, thereby creating a solution in the material cell, which then diffused out of the material cell. These steps need adequate time to achieve a balance. The effect of different extraction times on TCBL polysaccharide yield is shown in Figure 3. The TCBL polysaccharide yield increased for extraction times of 20 to 30 min. When the extraction time was 30 min, the highest yield of polysaccharides was 4.76%. However, the extraction time continued to progress, and the polysaccharide yield from TCBL decreased slightly. As the extraction time progressed, it changed the chemical character and destroyed the structure of polysaccharides or other water-soluble impurities, which had to be leached at a higher temperature for a longer time; this affected the extraction rate of polysaccharides. Therefore, 30 min was chosen as the enzymatic/ultrasound treatment time.

#### 3.2.3. Effect of Different Material to Liquid Ratios on the TCBL Polysaccharide Yield

**Figure 4:**
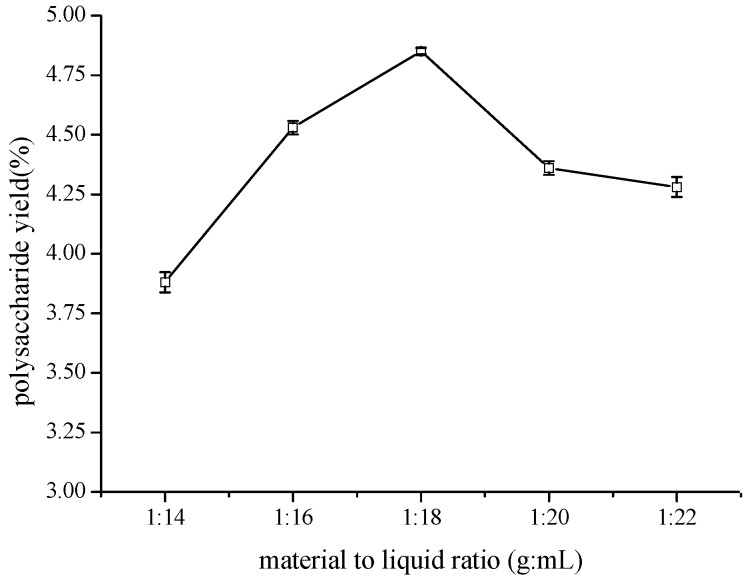
Effect of different material to liquid ratios on the polysaccharide yield (w/w dry crushed material %) from TCBL.

A quantity of 3 g of crushed material from TCBL was extracted in different pectinase concentration solutions under ultrasonic treatment of 144 W at 50 °C for 30 min. The effect of different ratios of material to liquid on the yield of polysaccharide TCBL was investigated. The result is shown in Figure 4. It illustrates the effect of different ratios of material to liquid (1:14, 1:16, 1:18, 1:20, and 1:22 g·mL^−1^) on the TCBL polysaccharide yield. As can be seen in Figure 3, in a certain range of 1:14 to 1:18 g·mL^−1^ of the ratio of material to liquid, increasing the ratio of material to liquid could facilitate complete immersion of material into the liquid, accelerate mass transfer, and elevate the yield of polysaccharides from TCBL. When the ratio of material to liquid was 1:18, the highest yield of polysaccharides was 4.85%. However, when the ratio of material to liquid continued to increase, the TCBL polysaccharide yield decreased slightly. On the basis of this result, a 1:18 ratio of raw material to liquid was chosen as the optimum ratio for subsequent experiments. Therefore, the best ratio of material to liquid was 1:18.

#### 3.2.4. Effect of Enzyme Concentration on TCBL Polysaccharide Yield

**Figure 5:**
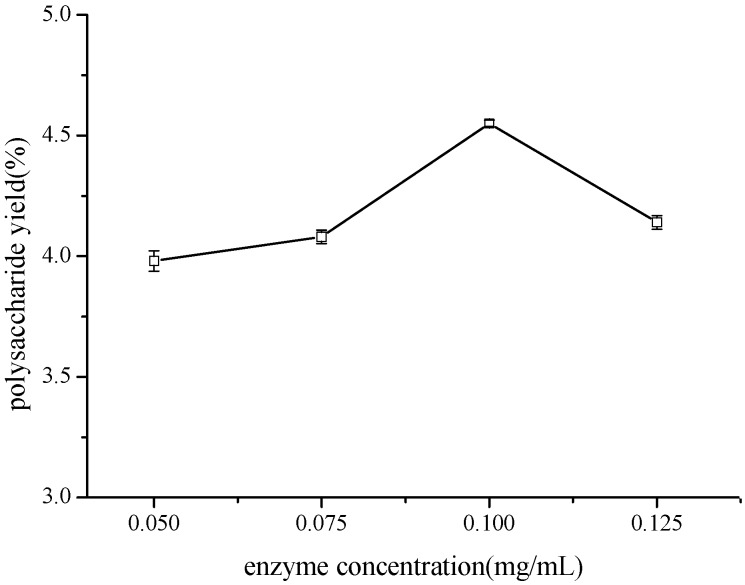
Effect of enzyme concentration on polysaccharide yield (w/w dry crushed material %) from TCBL.

Under a 1:18 ratio of material to liquid at 50 °C, the effect of different enzyme concentrations (0.05, 0.075, 0.100, and 0.125 mg·mL^−1^) on the yield of polysaccharides from TCBL was investigated by 144 W ultrasound treatment for 40 min. The results are shown in Figure 5.

The result demonstrated that the yield of TCBL polysaccharides increased with the increase of enzyme concentration between 0.050 and 0.100 mg·mL^−1^. When the enzyme concentration was 0.100 mg·mL^−1^, the highest yield of polysaccharides was 4.55%. However, when the enzyme concentration continued to decrease, the yield of polysaccharides from TCBL became small. As the enzyme concentration increased (<0.100 mg·mL^−1^), the rate of enzymatic hydrolysis of the substrate increased, and the polysaccharide yield increased gradually. When the enzyme concentration reached 0.100 mg·mL^−1^, the amount of enzyme added and the substrate concentration were most suitable, and the polysaccharide yield was the highest. When the enzyme concentration was higher than 0.100 mg·mL^−1^, there was no excess substrate bound to the active site of the enzyme, and, therefore, the extracted polysaccharide would bind to the enzyme. As a result, the polysaccharide would be hydrolyzed and the yield would decrease.

### 3.3. Response–Surface Methodology of TCBL Polysaccharide Extraction Technology by Enzymes and Ultrasound-Assisted Coupled Extraction (EUCE)

#### 3.3.1. Response–Surface Methodology Experimental Design and Results

Based on the single-factor experimental results of polysaccharide extraction technology by EUCE, the experimental data were analyzed by response–surface methodology. The experimental results were obtained, as shown in Table 2. The results of Table 2 are fitted by multiple linear regression, and the quadratic polynomial regression equation is as follows:

Y = 4.71 + 0.12X_1_ + 0.014X_2_ + 0.085X_3_ − 0.023X_4_ − 0.06X_1_X_2_ − 0.092X_1_X_3_ − 0.068X_1_X_4_ + 0.14X_2_X_3_ − 0.03X_2_X_4_ + 5 × 10^−3^X_3_X_4_ − 0.28X_1_^2^ − 0.20X_2_^2^ − 0.15X_3_^2^ − 0.18X_4_^2^. (4)

#### 3.3.2. Response–Surface Methodology Experimental Results

In order to confirm the validity of the regression equation and the influence of various factors on the yield of TCBL polysaccharide, the regression model was analyzed by variance analysis. The results are shown in Table 3.

Table 3 shows that the *p*-value of the model was less than 0.0001, implying the regression equation was significant. The *p*-value (0.1055) of lack of fit indicates that the model was not significant. R^2^ was greater than 0.9, demonstrating that the model fit well. Therefore, the response–surface method can be used to determine the optimal extraction process of polysaccharides from TCBL.

**Figure 6:**
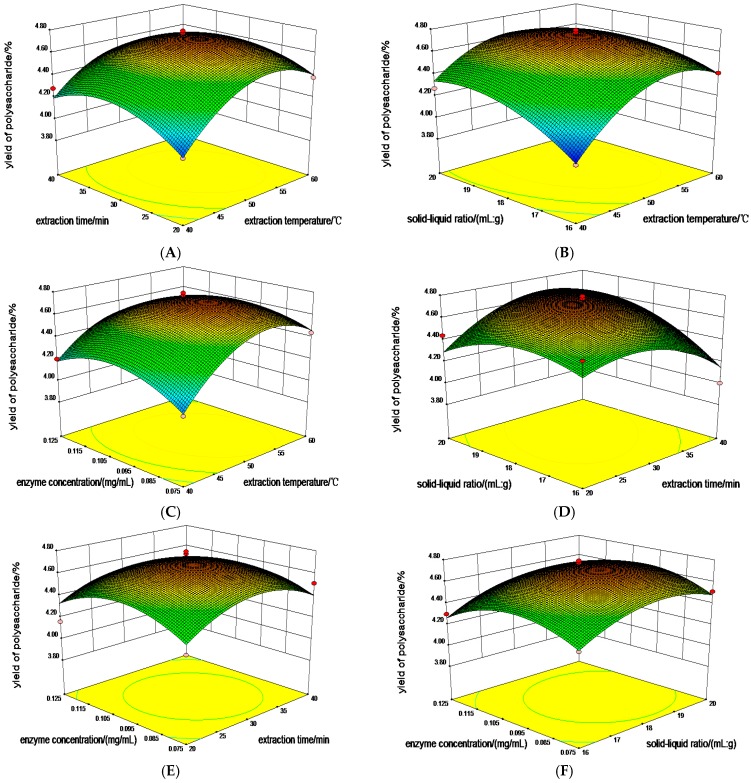
Response–surface plots for processing parameters (**A**) extraction time and temperature, (**B**) solid-liquid ratio and extraction temperature, (**C**) enzyme concentration and extraction temperature, (**D**) solid-liquid ratio and extraction time, (**E**) enzyme concentration and extraction time, and (**F**) enzyme concentration and solid-liquid ratio on polysaccharide yield.

The regression model (*p* < 0.0001) shows a significant effect on the yield of polysaccharides from TCBL. From Table 3, it can be seen that X_1_ and X_3_ were less than 0.05. This illustrates that the extraction temperature and the ratio of material to liquid had a significant effect on the yield of polysaccharides from TCBL, but the extraction time and enzyme concentration were not significant. The main factors affecting the yield of polysaccharides were extraction temperature (X_1_) > the liquid ratio (X_3_) > enzyme concentration (X_4_) > extraction time (X_2_); X_1_X_2_, X_1_X_3_, X_1_^2^ < 0.0001. The results demonstrate that the interactive quadratic terms of extraction temperature and extraction time, the interactive quadratic terms of extraction temperature and material:liquid ratio, and the quadratic terms of extraction temperature had a significant effect on the yield. X_2_X_3_, X_2_^2^, X_3_^2^, and X_4_^2^ were all less than 0.05, as indicated by the interactive quadratic terms of extraction time and material:liquid ratio, the quadratic terms of extraction time, the material:liquid ratio, and the quadratic terms of extraction temperature, respectively. The second term in enzyme concentration equation had a significant effect on the yield of polysaccharide. The above analysis shows that the influence of various factors on the yield of polysaccharides is not a simple linear relationship but an interactive relationship. The response–surface plots are shown in Figure 6.

Figure 6 is a response–surface graph of an optimization experiment by the EUCE method. The graph shows the interaction between various factors on the yield of TCBL polysaccharide. If the surface of the response–surface graph is steeper, the influence of this factor on the yield of TCBL polysaccharides is greater. By response–surface software analysis, the predicted optimum extraction technology conditions of polysaccharides from TCBL were determined to be as follows: an extraction temperature of 51 °C an extraction time of 33 min, a ratio of material to liquid of 1:19 g·mL^−1^, enzyme concentration of 0.097 mg·mL^−1^, and a predicted yield of TCBL polysaccharide of 4.77%. To verify that the predicted yield of TCBL polysaccharide did not deviate from the practical value. Verification experiments were performed using modified optimal technology parameter values for maximal yield of TCBL polysaccharide, which were an extraction temperature of 51 °C, an extraction time of 33 min, a raw material to liquid ratio of 1:19 g·mL^−1^, and enzyme concentration of 0.1 mg·mL^−1^. The actual yield of TCBL polysaccharide was 4.78% ± 0.18% (n = 3), which was similar to the predicted value. The predictive model coincided with the actual experiment. The relative error between the yield of polysaccharides and the predicted value by response–surface software analysis was 0.1%. The optimum result of the response–surface was reliable.

#### 3.3.3. Determination of Total Polysaccharide Content in TCBL 

**Figure 7:**
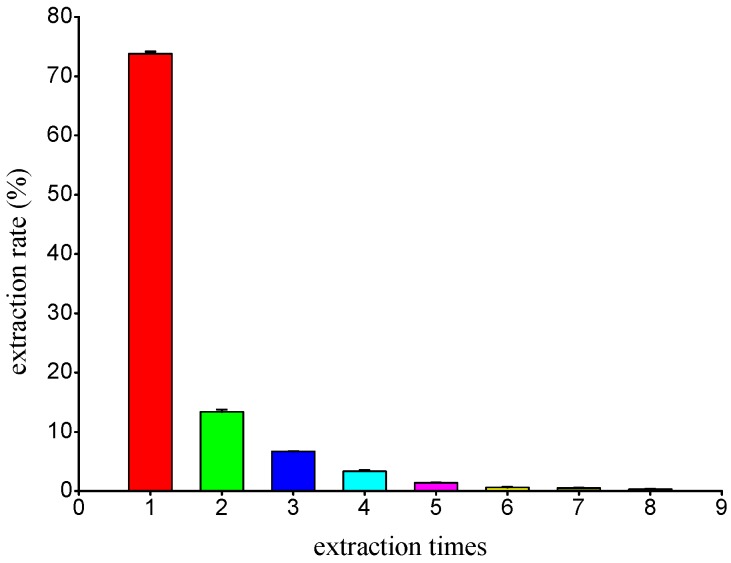
The relationship between extraction times and extraction rates of the TCBL polysaccharide.

The raw material of TCBL was extracted eight times according to the extraction technology conditions detailed in Section 3.3.2. The content of polysaccharides in each extract was determined by the anthrone–sulfuric acid method. The extraction rate of polysaccharide each time accounted for the total content of polysaccharides shown in Figure 7.

As shown in Figure 6, the polysaccharide content from TCBL extract was 73.8% in the first extraction and 0.33% in the eighth extraction. The total polysaccharide content of TCBL was calculated to be 8.88%, according to the polysaccharide content of extracts of all eight extraction times. After five extractions (repeated three times), the polysaccharide extraction rate was more than 98%. Therefore, this demonstrated TCBL polysaccharides could be almost completely extracted after five extractions.

### 3.4. Analysis Results of Monosaccharide Composition of Polysaccharides from TCBL

**Figure 8:**
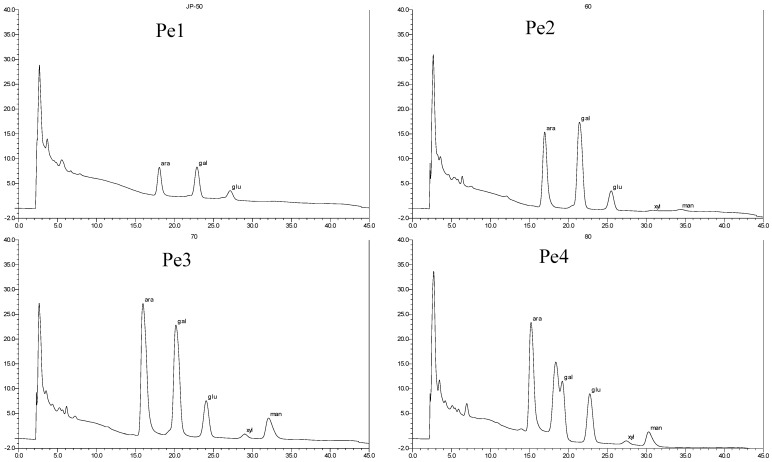
Monosaccharide composition of *Taxus* polysaccharides. Pe1: 50% alcohol-precipitated polysaccharide, Pe2: 60% alcohol-precipitated polysaccharide, Pe3: 70% alcohol-precipitated polysaccharide, and Pe4: 80% alcohol-precipitated polysaccharide.

The 50%, 60%, 70%, and 80% alcohol precipitation polysaccharides (Pe1, Pe2, Pe3, and Pe4) were hydrolyzed to monosaccharides with sulfuric acid. The analysis of monosaccharide compositions of four different polysaccharides from TCBL was carried out, and the results are shown in Figure 8.

The monosaccharide composition of polysaccharides from TCBL was obtained by comparing Figure 7 with standard monosaccharide, and the results are shown in Table 4. 

Analysis results illustrated in Table 4 indicate the polysaccharides from TCBL were mainly composed of arabinose, galactose, glucose, and a small amount of xylose and mannose. 

The ratio of monosaccharide composition of Pe1 of arabinose:galactose:glucose was about 2:2.5:1. The ratio of arab:gal:glu:xyl:man of Pe2 was 81:111:24:1:2. The ratio of arab:gal:glu:xyl:man of Pe3 was about 34:30:9:1:6. The ratio of arab:gal:glu:xyl:man of Pe4 was about 25:12:13:1:4. Therefore, the monosaccharide composition of Pe1 had no xylose and mannose. The Pe2 monosaccharide was mainly composed of arabinose, galactose, glucose, and a slight amount of xylose and mannose. The Pe3 and Pe4 monosaccharides were composed of arabinose, glucose, galactose, and small amounts of mannose and xylose.

### 3.5. α-Glucosidase Inhibitory Activity 

α-Glucosidase is a type of hydrolase that hydrolyzes the α-1,4-glycosidic linkage of a sugar and converts it to glucose. It has been reported that α-glucosidase inhibitors can effectively delay and alleviate the time and progression of postprandial blood glucose elevation in diabetic patients; therefore, studying the activity of α-glucosidase inhibitors can help control the development of diabetes and the occurrence of complications. [29,30].

Four purified polysaccharides were assayed in a range of concentrations (10.00~120.00 µg·mL^−1^). Acarbose was used as a positive control. Data are presented as mean ± SD. RSD < 5%.

**Figure 9:**
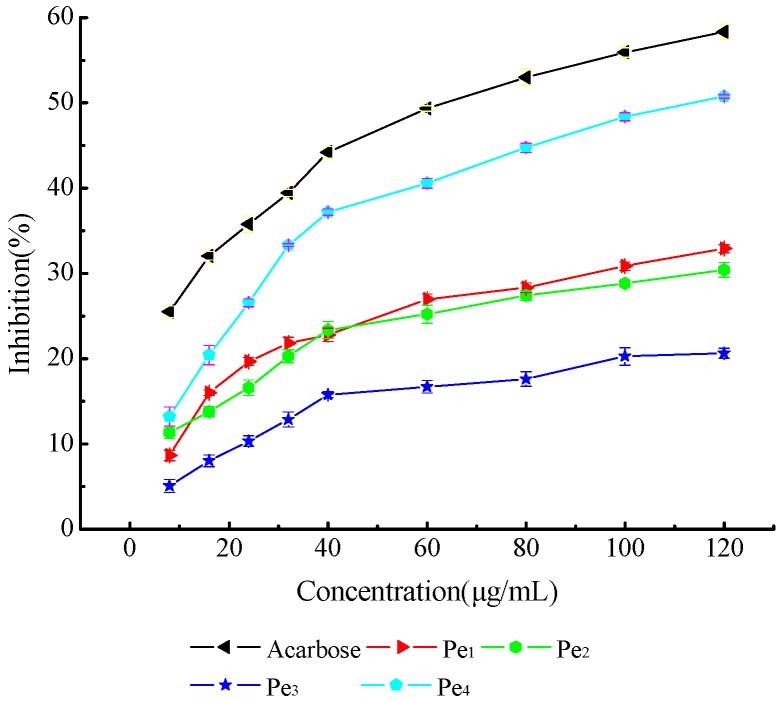
Dose–response curves of α-glucosidase inhibitory activity of four purified polysaccharides (Pe1, Pe2, Pe3 and Pe4).

α-Glucosidase was effectively inhibited by four purified polysaccharides (Pe1, Pe2, Pe3 and Pe4) from TCBL (Figure 8). The α-glucosidase inhibitory activity of four purified polysaccharides from TCBL was slightly lower than that of acarbose, which was used as a positive control sample. Figure 9 illustrated a dose-dependent inhibitory effect of α-glucosidase. The purified Pe4 polysaccharide had the highest α-glucosidase inhibitory activity among the four purified polysaccharides. The IC_50_ value of Pe4-inhibiting α-glucosidase was 114.0 µg·mL^−1^; and acarbose was 63.7 µg·mL^−1^. We also measured the molar mass of Pe4, which was 6536 g/mol. The IC_50_ of Pe 4 was 17.4 µM, while that of acarbose was 98.7 µM. The study suggested that the polysaccharides from TCBL had the ability to inhibit α-glucosidase activity. The 𝛼-glucosidase activities were inhibited so that glucose absorption and postprandial hyperglycemia also decreased [31]. This affected serum glucose production, which is associated with type 2 diabetes. Therefore, the polysaccharides from TCBL potently inhibited α-glucosidase activity associated with type 2 diabetes.

### 3.6. Antitumor Activity 

Antitumor activity assays were performed using the MTT method. The assay principle is that the succinate dehydrogenase in the living cell’s mitochondria can decrease exogenous MTT to a blue-violet crystal. The crystal is insoluble in water but soluble in DMSO. The crystal is dissolved with DMSO as a solvent and measured at a certain absorbance value at a certain wavelength. Succinate dehydrogenase is not produced in dead cells. 

**Figure 10:**
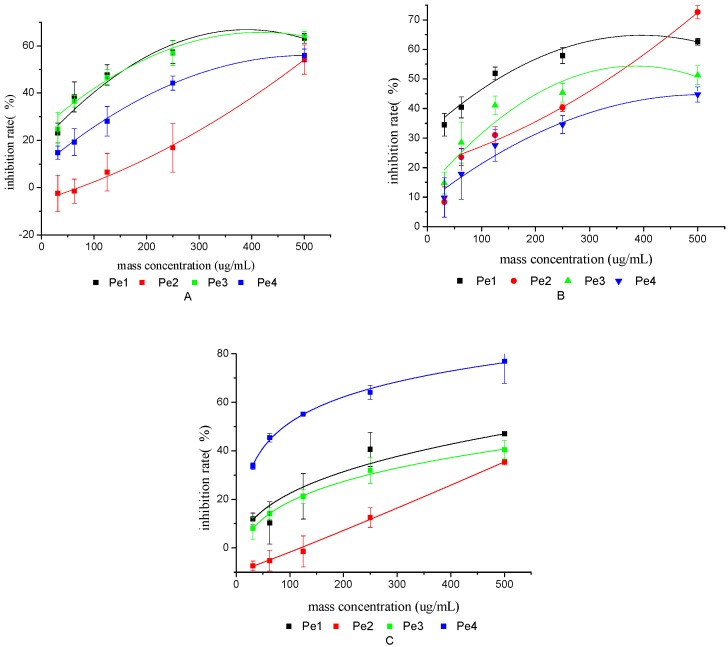
Results of antitumor activities of polysaccharides from TCBL against MCF7 cells (**A**), HepG2 cells (**B**), and Hela cells (**C**).

The ability of four purified polysaccharides (Pe1, Pe2, Pe3, and Pe4) from TCBL to inhibit three types of cancer cells (MCF7, Hela, and HepG2) is shown in Figure 10. The IC_50_ values of the antitumor activity of the four purified polysaccharides against the three types of cancer cells are shown in Table 5.

As shown in Figure 10 and Table 5, the 50% alcohol-precipitated polysaccharide (Pe1) showed a better inhibitory activity against human breast cancer cells (MCF7) and human hepatoma cells (HepG2), with IC_50_ values of 169.0 and 132.0 µg·mL^−1^, respectively. The 80% alcohol-precipitated polysaccharide (Pe4) had a good inhibitory effect on human cervical cancer cells (Hela), and the half inhibition rate was the lowest among the four purified polysaccharides. Its IC_50_ value was 89.9 µg·mL^−1^ (13.8 µM), and the IC_50_ value of the antitumor drug etoposide was 21.2 µg·mL^−1^ (36.0 µM). Therefore, the 80% alcohol-precipitated polysaccharide (Pe4) can be used as a potential natural antitumor bioactive drug against human cervical cancer cells (Hela).

## 4. Conclusions

This research characterized the optimum extraction technology of polysaccharides from TCBL using a low-temperature and high-efficiency enzyme and ultrasound-assisted coupled extraction (EUCE) method for the first time. Optimal technology parameters were ascertained as follows: an extraction temperature of 51 °C, an extraction time of 33 min, a ratio of material to liquid of 1:19 (g:mL), and an enzyme concentration of 0.10 mg·mL^−1^. Under the optimized conditions, the polysaccharide yield from TCBL obtained by EUCE was 4.78% ± 0.18%. Moreover, the polysaccharide yield by EUCE was the highest compared to the HWRE, UAE and EE extraction methods. Therefore, the EUCE method to extract polysaccharides from *Taxus cuspidata* branches and leaves was the superior extraction method.

The four purified polysaccharides (Pe1, Pe2, Pe3, and Pe4) were obtained after the crude polysaccharide extracts of TCBL were separated and purified by the macroporous adsorption resin method; 50%, 60%, 70%, and 80% of alcohol precipitation methods; and dialysis and DEAE cellulose column chromatography. The four purified polysaccharides from TCBL were characterized by HPIC analysis. The results demonstrated that the monosaccharide compositions of polysaccharides from TCBL are mainly composed of arabinose, galactose, glucose, and a small amount of xylose and mannose. Antidiabetic activity and antitumor activity of polysaccharides from TCBL were evaluated using in vitro assays. Among the four purified polysaccharides from TCBL, purified Pe4 demonstrated good inhibitory activity on α-glucosidase, and its IC_50_ value was 123.0 µg·mL^−1^. Pe1 had the higher antitumor capacity against MCF7 cells and HepG2 cells, with IC_50_ values of 169.0 µg·mL^−1^ and 132.0 µg·mL^−1^. Pe4 had the greatest inhibitory effect on Hela cells, and its IC_50_ value was 89.9 µg·mL^−1^. 

This research demonstrated Pe4 polysaccharide had a good α-glucosidase inhibitory activity and antitumor capacity against human cervical cancer cells (Hela). Therefore, polysaccharides from TCBL can be used as the beneficial sources of potential inhibitors of type 2 diabetes and human cervical cancer activity. 

## Figures and Tables

**Table 1:** Comparison of extraction methods of polysaccharides from *Taxus cuspidata* branches and leaves (TCBL).

Extraction Methods	Polysaccharide yield (w/w dry material %) mean ± SD
hot water reflux extraction (HWRE)	3.71 ± 0.05
ultrasonic-assisted extraction (UAE)	3.89 ± 0.07
enzyme extraction (EE)	4.27 ± 0.01
enzyme and ultrasound-assisted coupled extraction (EUCE)	4.47 ± 0.02

**Table 2:** Response–surface methodology experimental design and results of TCBL polysaccharide extraction technology by enzyme and ultrasound-assisted coupled extraction (EUCE).

Run	X_1_ (Extraction Temperature, °C)	X_2_ (Extraction Time, min)	X_3_ (Ratio of Raw Material to Liquid, g:mL)	X_4_ (Enzyme Concentration, mg·mL^−1^)	TCBL Polysaccharide Yield (%)
1	50	40	1:16	0.100	4.00
2	60	30	1:20	0.100	4.34
3	50	20	1:16	0.100	4.54
4	50	30	1:20	0.075	4.51
5	50	30	1:20	0.125	4.52
6	50	40	1:20	0.100	4.47
7	40	30	1:18	0.075	4.07
8	60	20	1:18	0.100	4.38
9	50	30	1:18	0.100	4.77
10	50	30	1:18	0.100	4.79
11	50	30	1:18	0.100	4.68
12	50	20	1:18	0.075	4.23
13	50	20	1:18	0.125	4.16
14	50	40	1:18	0.125	4.32
15	50	40	1:18	0.075	4.51
16	60	30	1:18	0.075	4.44
17	50	30	1:18	0.100	4.68
18	50	30	1:18	0.100	4.64
19	50	30	1:16	0.125	4.30
20	40	30	1:16	0.100	3.97
21	60	40	1:18	0.100	4.38
22	60	30	1:18	0.125	4.30
23	40	30	1:18	0.125	4.20
24	50	20	1:20	0.100	4.44
25	40	30	1:20	0.100	4.27
26	40	40	1:18	0.100	4.28
27	50	30	1:16	0.075	4.31
28	40	20	1:18	0.100	4.04
29	60	30	1:16	0.100	4.41

**Table 3:** Results of response–surface variance analysis of TCBL polysaccharide yield.

Source ^a^	Sun of Squares	Degree of Freedom	Mean Square	F-Value	*p*-Value	Significance ^b^
model	1.16	14	0.083	6.64	<0.0001	**
X_1_	0.17	1	0.17	13.47	0.0025	*
X_2_	2.41 × 10^−^^3^	1	2.41 × 10^−^^3^	0.19	0.6671	
X_3_	0.087	1	0.087	6.95	0.0196	*
X_4_	6.08 × 10^−^^3^	1	6.08 × 10^−^^3^	0.49	0.4967	
X_1_X_2_	0.014	1	0.014	1.15	<0.0001	**
X_1_X_3_	0.034	1	0.034	2.74	<0.0001	**
X_1_X_4_	0.018	1	0.018	1.46	0.2468	
X_2_X_3_	0.081	1	0.081	6.51	0.023	*
X_2_X_4_	3.60 × 10^−^^3^	1	3.60 × 10^−^^3^	0.29	0.5996	
X_3_X_4_	1.00 × 10^−^^4^	1	1.00 × 10^−^^4^	8.0 × 10^−^^3^	0.9299	
X_1_^2^	0.5	1	0.5	40.44	<0.0001	**
X_2_^2^	0.25	1	0.25	19.8	0.0005	*
X_3_^2^	0.15	1	0.15	12.32	0.0035	*
X_4_^2^	0.21	1	0.21	16.88	0.0011	*
Residual	0.17	14	0.012			
Lack of fit	0.16	10	0.016	3.79	0.1055	
Pure error	0.017	4	4.17 × 10^−^^3^			
Total	1.34	28				

a X_1_: extraction temperature, X_2_: extraction time, X_3_: ratio of raw material to liquid, X_4_: enzyme concentration; b * Significant difference (0.01 < *p* < 0.05); ** Significant difference (*p* < 0.01).

**Table 4:** Analysis results of monosaccharide composition of polysaccharides from TCBL.

4 Purified Polysaccharides	Percentage of Monosaccharide (%)				
	Arabinose	Galactose	Glucose	Xylose	Mannose
Pe1	37.07	45.11	17.82	—	—
Pe2	36.93	50.87	10.88	0.46	0.86
Pe3	42.33	37.41	10.95	1.26	8.05
Pe4	45.50	21.39	23.35	1.83	7.93

**Table 5:** The IC_50_ values of the antitumor activity of the four purified polysaccharides against the three types of cancer cells.

4 Purified Polysaccharides	IC_50_ µg·mL^−1^		
	MCF7	Hela	HepG2
Pe1	169.0	364.9	132.0
Pe2	465.9	>500	347.7
Pe3	252.8	>500	343.9
Pe4	376.2	89.9	>500
etoposide	2.8	21.2	25.3
